# Effect of Angle Opening Parameters on Corneal Endothelial Cell Density and Intraocular Pressure after Posterior Chamber Phakic Intraocular Lens Implantation

**DOI:** 10.3390/jcm9092704

**Published:** 2020-08-21

**Authors:** Kazutaka Kamiya, Wakako Ando, Tatsuhiko Tsujisawa, Masahide Takahashi, Nobuyuki Shoji

**Affiliations:** 1 Visual Physiology, School of Allied Health Sciences, Kitasato University, Kanagawa 252-0373, Japan; 2 Department of Ophthalmology, School of Medicine, Kitasato University, Kanagawa 252-0374, Japan; wakako@kitasato-u.ac.jp (W.A.); fighting_tatsuhiko@yahoo.co.jp (T.T.); zavide96@gmail.com (M.T.); nshoji@kitasato-u.ac.jp (N.S.)

**Keywords:** angle open distance, hole ICL, endothelial cell density, intraocular pressure, ICL, phakic IOL

## Abstract

This study aimed to investigate the time courses of angle opening parameters and the relationships of these with the corneal endothelial cell density (ECD) and the intraocular pressure (IOP) after posterior chamber phakic intraocular lens (Visian ICL^TM^, STAAR Surgical) implantation. We evaluated 116 eyes of 59 consecutive patients (mean age ± standard deviation, 34.0 ± 8.8 years) who underwent V5 implantable collamer lens (ICL) implantation. Preoperatively and 1 day, 1 week, and 1, 3, and 18 months postoperatively, we quantitatively measured the angle opening distance at 500 µm (AOD500), the trabecular-iris space area (TISA500), and the trabecular iris angle (TIA500), using anterior segment optical coherence tomography (CASIA 2, Tomey), and assessed the relationships of these measurements with ECD and IOP in ICL-implanted eyes. All angle parameters (AOD500, TISA500, and TIA500) significantly decreased 1 day postoperatively but remained stable thereafter. At 18 months postoperatively, we found no significant correlations of the angle parameters with ECD (Pearson correlation coefficient *r* = −0.108, *p* = 0.249 for AOD500; *r* = −0.162, *p* = 0.083 for TISA500; *r* = −0.022, *p* = 0.815 for TIA500) or between the angle parameters and IOP (*r* = −0.106, *p* = 0.256 for AOD500; *r* = −0.021, *p* = 0.826 for TISA500; *r* = −0.018, *p* = 0.850 for TIA500). The angle opening metrics significantly decreased immediately after ICL implantation but remained stable thereafter. Narrowing of the angle did not significantly affect ECD or IOP in ICL-implanted eyes during the 18-month postoperative period.

## 1. Introduction

The EVO Visian Implantable Collamer Lens (ICL^TM^, STAAR Surgical, Monrovia, CA, USA), a posterior chamber phakic intraocular lens, has become widely accepted as an effective treatment of moderate to high ametropia, over a long period of time [1,2,3,4]. However, angle closure and subsequent intraocular pressure (IOP) rise can occur when a larger implantable collamer lens (ICL) is selected, and cataract formation or toric ICL rotation can occur when a smaller ICL is selected. Therefore, effective ICL sizing is still challenging in daily practice, and thus can be considered as one of the main issues of ICL surgery safety.

Swept source anterior segment optical coherence tomography (AS-OCT) has been commercially available in a clinical setting and provides more accurate measurements of the anterior segment metrics, such as anterior chamber depth, angle-to-angle distance, and angle opening parameters, than conventional ultrasound biomicroscopy (UBM) [5]. Angle opening metrics are clinically useful to grasp the actual opening status of the angle, since these changes may lead to corneal endothelial cell density (ECD) loss as well as to a rise in IOP. However, the detailed time course of angle opening metrics has not been fully elucidated in ICL-implanted eyes, especially in the early postoperative period. Moreover, to the best of our knowledge, the effects of these angle metrics on ECD and IOP levels have so far not been investigated in such eyes. This may give us intrinsic insights on the clinical implication of the angle opening after ICL implantation. The goal of the present study was twofold: (1) to assess the time course of changes in the angle opening parameters and (2) to investigate the relationships of (1) with the ECD and the IOP in eyes undergoing ICL implantation.

## 2. Patients and Methods

### 2.1. Study Population

**Figure 1:**
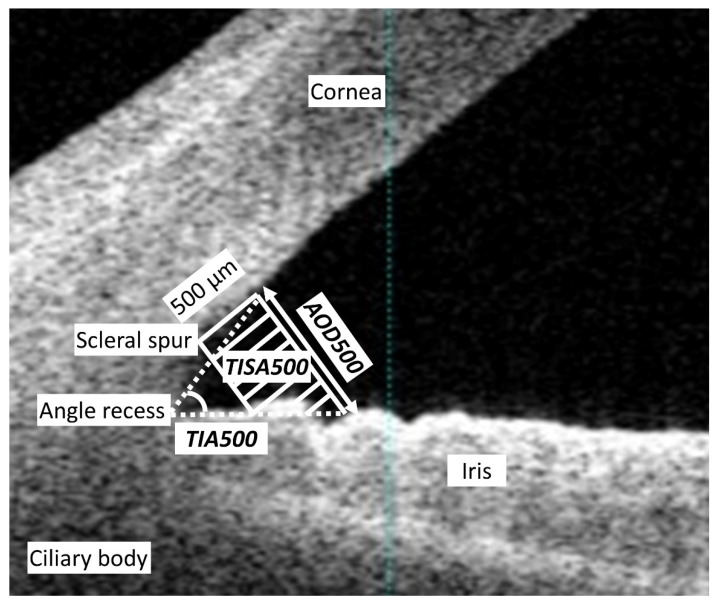
A schematic diagram of the angle opening parameters. AOD500 is the distance between the posterior cornea surface and the anterior iris surface measured on a line perpendicular to the trabecular meshwork at 500 µm from the scleral spur. TISA500 is the surface area of a trapezoid with the following boundaries: anteriorly, the AOD500 from the scleral spur; posteriorly, a line drawn from the scleral spur perpendicular to the plane of the inner scleral wall to the iris; superiorly, the inner corneoscleral wall; and inferiorly, the iris surface. TIA500 is a trabecular iris angle at 500 µm from the scleral spur.

We registered the study protocol with the University Hospital Medical Information Network Clinical Trial Registry (000040197). We evaluated 116 eyes of 59 consecutive patients (25 men and 34 women) who underwent ICL implantation (ICL model: V5 with a central hole) for the correction of moderate to high myopia and myopic astigmatism and who completed a 18-month follow-up at Kitasato University Hospital in this series. The inclusion criteria for ICL surgery at our institution were as follows: unsatisfactory correction with spectacles or contact lenses, 20 ≤ age ≤ 50 years, stable refraction for at least 3 months, −3.00 to −14.0 diopters (D) of myopia with astigmatism of 3 D or less, anterior chamber depth ≥ 2.8 mm, endothelial cell density ≥ 1800 cells/mm^2^, and no history of ocular surgery, corneal degeneration, cataract, glaucoma, or uveitis. We excluded keratoconic eyes from this study by using the screening test equipped with AS-OCT (CASIA 2^TM^, Tomey, Nagoya, Japan). The sample size in the present study offered 89.3% statistical power at the 5% level in order to detect a 0.3-mm difference in the AOD500, when the standard deviation (SD) of the mean difference was 1.0 mm. Preoperatively and 1 day, 1 week, and 1 and 3 months postoperatively, we measured the logarithm of the minimum angle of resolution (logMAR) of uncorrected and corrected visual acuities, the manifest spherical equivalent, IOP with a non-contact tonometer (KT-500^TM^, Kowa, Tokyo, Japan), and angle parameters such as the angle open distance at 500 µm (AOD500), the trabecular-iris space area at 500 µm (TISA500), and the trabecular iris angle at 500 µm (TIA500), at the horizontal plane, automatically obtained by AS-OCT under bright light conditions (500 lux). AOD500 was defined as the distance between the posterior cornea surface and the anterior iris surface measured on a line perpendicular to the trabecular meshwork at 500 µm from the scleral spur. TISA500 was defined as the surface area of a trapezoid with the following boundaries: anteriorly, the AOD500 from the scleral spur; posteriorly, a line drawn from the scleral spur perpendicular to the plane of the inner scleral wall to the iris; superiorly, the inner corneoscleral wall; and inferiorly, the iris surface. TIA500 was defined as a trabecular iris angle at 500 µm from the scleral spur (Figure 1). The average values at the nasal and temporal angles were utilized for statistical analysis. Preoperatively and 18 months postoperatively, we also systematically measured the ECD with a non-contact specular microscope (EM-3000^TM^, Tomey, Nagoya, Japan). Patients were asked to blink just before starting the AS-OCT and ECD measurements. After we first checked the image quality, we selected one examination with a high image quality. Written informed consent for the ICL surgery was obtained from all patients. This retrospective review of the clinical charts was approved by the Institutional Review Board at Kitasato University Hospital (B18-222) and followed the tenets of the Declaration of Helsinki. Our Institutional Review Board waived the requirement for informed consent for this retrospective study.

### 2.2. Lens Size Selection and Power Calculation

We selected the appropriate ICL size by referring to the manufacturer’s nomogram based on the horizontal corneal diameter and anterior chamber depth using AS-OCT. We also determined the ICL power using an online calculator system provided by the manufacturer, which was based on a modified vertex formula [6,7].

### 2.3. Surgical Procedure

Detailed surgical procedures have been described previously in our studies [6,7]. In brief, on the day of surgery, dilating and topically anesthetic agents were administered. A model V5 ICL was implanted through a 3-mm temporal clear corneal incision after placement of a viscosurgical device in the anterior chamber. The ICL was inserted into the posterior chamber, the viscosurgical device was replaced with balanced salt solution, and a miotic agent was administered. We topically used steroidal (0.1% betamethasone) and antibiotic (1.5% levofloxacin) medications 4 times daily for 1 week, with the dose being reduced gradually thereafter.

### 2.4. Statistical Analysis

We conducted statistical analyses by using commercially available statistical software (Bellcurve for Excel, Social Survey Research Information Co, Ltd., Tokyo, Japan). A one-way analysis of variance (ANOVA) was utilized for the analysis of the time course of changes, with the Dunnett test being employed for multiple comparisons. The Pearson correlation coefficient was utilized to assess the relationships of the two variables. The paired t-test was used to compare the preoperative and postoperative data. Unless otherwise indicated, the results are expressed as mean ± SD, and a value of *p* < 0.05 was considered statistically significant.

## 3. Results

Table 1 summarizes the preoperative and postoperative demographics of the study population. All surgeries were uneventful. The ECD changed from 2815 ± 220 cells/mm^2^ (range of 2229 to 3351 cells/mm^2^) preoperatively to 2775 ± 226 cells/mm^2^ (2306 to 3360 cells/mm^2^) at 18 months postoperatively (*p* = 0.173). The mean ECD loss was 1.5% ± 5.8% (−10.2% to 12.7%). The mean IOP was 14.0 ± 3.0 mmHg at 18 months postoperatively. We found no significant complications, such as cataract formation, significant ECD loss (>20%), significant IOP rise (>22 mmHg), pigment dispersion glaucoma, pupillary block, uveitis, or any other vision-threatening adverse event at any time in this series.

**Figure 2:**
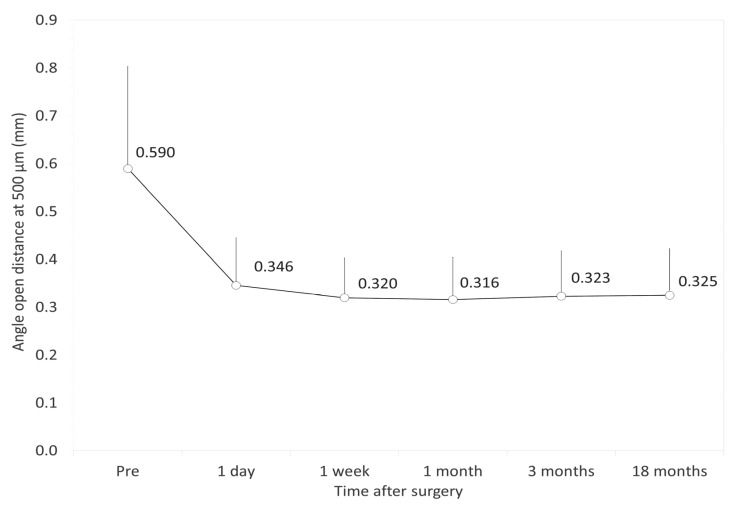
Time course of the angle opening distance at 500 µm (AOD500) after implantable collamer lens (ICL) implantation. The AOD500 was significantly decreased 1 day postoperatively and subsequently stabilized over time. Bar represents standard deviation.

**Figure 3:**
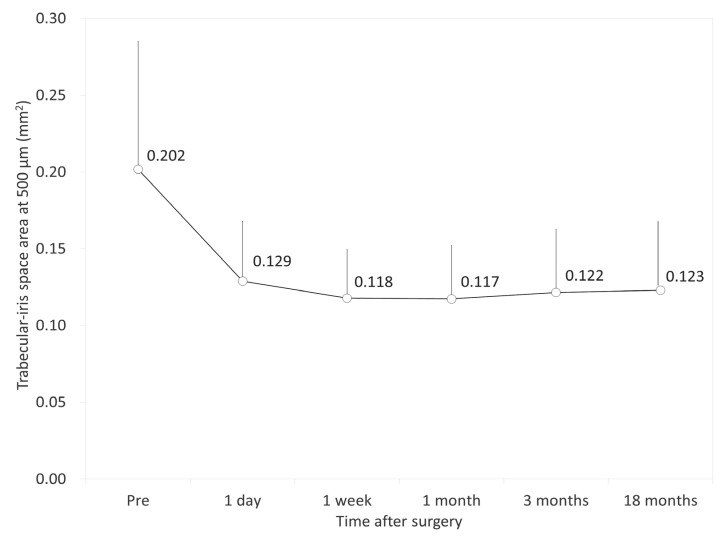
Time course of trabecular-iris space area at 500 µm (TISA500) after implantable collamer lens (ICL) implantation. The TISA500 was significantly decreased 1 day postoperatively and subsequently stabilized over time. Bar represents standard deviation.

**Figure 4:**
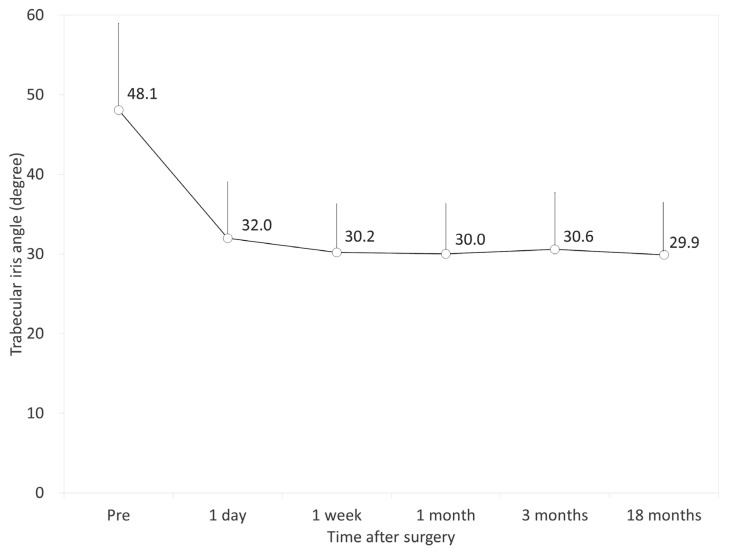
Time course of trabecular iris angle at 500 µm (TIA500) after implantable collamer lens (ICL) implantation. The TIA500 was significantly decreased 1 day postoperatively and subsequently stabilized over time. Bar represents standard deviation.

Figure 2, Figure 3 and Figure 4 show the time courses of AOD500, TISA500, and TIA500, respectively. The variance of the data was statistically significant (*p* < 0.001, one-way ANOVA). Multiple comparisons demonstrated significant differences between measurements made preoperatively and 1 day, 1 week, 1 month, 3 months, and 18 months postoperatively (*p* < 0.001, Dunnett test). On the other hand, they demonstrated no significant differences between the postoperative measurements. All angle opening parameters (AOD500, TISA500, and TIA500) significantly decreased 1 day postoperatively but remained stable during the 3-month observation period. Table 2 shows the relationships of the angle opening metrics with the ECD and the IOP. At 18 months postoperatively, we found no significant association between the angle parameters and the ECD (Pearson correlation coefficient *r* = −0.108, *p* = 0.249 for AOD500; *r* = −0.162, *p* = 0.083 for TISA500; *r* = −0.022, *p* = 0.815 for TIA500) or between the angle parameters and the IOP (*r* = −0.106, *p* = 0.256 for AOD500; *r* = −0.021, *p* = 0.826 for TISA500; *r* = −0.018, *p* = 0.850 for TIA500). We also found no significant association between the angle parameters and the ECD loss (*r* = −0.010, *p* = 0.918 for AOD500; *r* = −0.027, *p* = 0.772 for TISA500; *r* = 0.014, *p* = 0.881 for TIA500).

## 4. Discussion

In the current study, our findings showed that all angle opening parameters (AOD500, TISA500, and TIA500) significantly decreased immediately after ICL surgery, but the parameters remained unchanged thereafter. It is indicated that ICL surgery induces a significant narrowing of the angle, but these changes subsequently stabilize with no further narrowing during an 18-month observation period. Our findings also demonstrated that there were no significant correlations of any of the angle opening parameters with ECD or IOP after ICL surgery. It is suggested that the degree of the angle opening did not directly influence ECD or IOP in daily practice when we selected the appropriate ICL size according to the manufacturer’s instructions. As far as we can ascertain, this is the first published study to assess the relationships of angle opening metrics with ECD and IOP in current ICL-implanted eyes. We believe that this information will be helpful for understanding the possible role of the angle opening on corneal ECD loss and IOP rise in eyes undergoing ICL implantation in a clinical setting.

Previous studies on angle opening measurements in eyes undergoing ICL implantation are summarized in Table 3 [8,9,10,11,12,13,14,15]. With regard to UBM measurements, Chung et al. demonstrated that AOD500 and TIA were significantly smaller than preoperative values by 41.5% and 31.8%, respectively, 1 month postoperatively and that no significant progressive changes were observed thereafter [8]. Wang et al. showed that there were significant decreases in AOD500 and TIA after ICL implantation [9]. Cao et al. also demonstrated that AOD500 measured with the UBM was 0.32 ± 0.15 mm and that the percentages of eyes with TIA greater than 30°, between 21° and 30°, between 11° and 20°, and smaller than 10° were 29.1%, 50.0%, 11.6%, and 9.3%, respectively [10]. Lim et al. showed that the mean ratios of reduction of AOD500 and TIA were 27.32% and 28.41%, respectively, after ICL implantation in eyes with low anterior chamber depth (<2.8 mm) [11]. Li et al. mentioned that the postoperative AOD500 and TIA showed a statistical reduction in ICL-V4c-implanted eyes having primary iris and/or ciliary body cysts [15]. With regard to AS-OCT measurements, Fernández-Vigo et al. reported that considerable angle narrowing was detected 1 month postoperatively and that this narrowing remained stable at 3 months after ICL V4c implantation [12]. They also described a narrowing of the angle of 39% to 45% and no further narrowing beyond 1 month in the nasal, temporal, and inferior quadrants after ICL implantation [13]. Coskunseven et al. stated that AOD500, TISA500, and TIA500 significantly decreased 1 year after ICL implantation for the correction of hyperopic astigmatism [14]. Although the ICL model, observation period, and measurement device were different among previous and current studies, the previous findings of UBM and AS-OCT measurements are in good agreement with our findings that ICL surgery induced a significant angle narrowing and that this narrowing was unprogressive over time.

There were some limitations to our study. First, this study was conducted in a retrospective fashion. Second, the follow-up time was set to 3 months, since patients who are satisfied with visual outcomes tend to be lost during routine follow-ups. Last, we included both eyes in most cases in this study; however, only one eye should be used for statistical analysis. We confirmed similar results even when only one eye was randomly chosen from each patient, indicating that the bilateral issue was not particularly severe in the current study. However, based on the fact that there are inter-eye correlations and that most statistical tests assume that the observations are independent, it should be noted that it will increase the chance of a type I error when including bilateral data of the same patient. Generalized estimating equations as well as random effects models will be beneficial for the analysis of bilateral data to control for inter-eye correlation [16].

## 5. Conclusions

In summary, we found that all angle opening metrics significantly decreased immediately after ICL surgery, but they did not significantly change thereafter. Additionally, we found no significant associations of angle opening parameters with ECD or IOP in ICL-implanted eyes. These findings indicate that ICL implantation induces a significant angle narrowing that becomes nearly stable over time and that the degree of the angle opening does not directly affect the ECD or the IOP in daily practice when an appropriate ICL size is selected.

## Figures and Tables

**Table 1:** Preoperative and postoperative demographics of the study population undergoing implantable collamer lens implantation.

Demographic	Preoperative	Postoperative (18 months)	*p*-Value
Age (years)	34.0 ± 8.8 (95%CI, 16.7 to 51.0)		
Gender (male:female)	25:34		
Manifest spherical refraction (D)	−7.20 ± 3.62 (95%CI, −14.21 to −0.10)	0.01 ± 0.30 (95%CI, −0.57 to 0.59)	<0.001
Manifest cylinder (D)	−0.80 ± 0.96 (95%CI, −2.67 to 1.08)	−0.17 ± 0.35 (95%CI, −0.85 to 0.52)	<0.001
UDVA (logMAR)	1.17 ± 0.40 (95%CI, 0.39 to 1.96)	−0.17 ± 0.13 (95%CI, −0.43 to 0.09)	<0.001
CDVA (logMAR)	−0.23 ± 0.08 (95%CI, −0.39 to −0.07)	−0.23 ± 0.09 (95%CI, −0.40 to −0.06)	0.586
Intraocular pressure (mmHg)	14.2 ± 2.6 (95%CI, 9.1 to 19.3)	14.0 ± 3.0 (95%CI, 8.1 to 19.9)	0.495
Endothelial cell density (cells/mm^2^)	2815 ± 220 (95%CI, 2384 to 3246)	2775 ± 226 (95%CI, 2331 to 3218)	0.173
AOD500 (mm)	0.59 ± 0.21 (95%CI, 0.17 to 1.01)	0.32 ± 0.09 (95%CI, 0.15 to 0.50)	<0.001
TISA500 (mm^2^)	0.20 ± 0.08 (95%CI, 0.04 to 0.37)	0.12 ± 0.05 (95%CI, 0.02 to 0.22)	<0.001
TIA500 (degree)	48.1 ± 10.9 (95%CI, 26.7 to 69.5)	29.9 ± 7.3 (95%CI, 15.6 to 44.3)	<0.001

D = diopter; CI = confident interval; logMAR = logarithm of the minimal angle of resolution; UDVA = uncorrected distance visual acuity; CDVA = corrected distance visual acuity; AOD500 = angle open distance at 500 µm; TISA500 = trabecular-iris space area at 500 µm; TIA500 = trabecular iris angle at 500 µm.

**Table 2:** The relationships of the angle opening parameters with the endothelial cell density (ECD) and the intraocular pressure (IOP).

Postoperative	ECD		IOP		Δ Change	ΔECD		ΔIOP	
(18 Months)	Correlation	*p*-Value	Correlation	*p*-Value	(Pre–Post)	Correlation	*p*-Value	Correlation	*p*-Value
AOD500	−0.108	0.249	−0.106	0.256	ΔAOD500	−0.109	0.246	0.151	0.106
TISA500	−0.162	0.083	−0.021	0.826	ΔTISA500	−0.076	0.418	0.155	0.097
TIA500	−0.022	0.815	−0.018	0.850	ΔTIA500	−0.074	0.430	0.020	0.833

**Table 3:** Previous studies reporting angle opening metrics in eyes undergoing implantable collamer lens implantation.

Author	Year	Eyes	Model	F/U	Device	AOD500 (µm)	TISA500 (mm^2^)	TIA (°)
Chung et al. [8]	2009	48	V4	2Y	UBM	327.7 ± 177.8	N.A.	24.2 ± 5.5
Wang et al. [9]	2011	30	V4	1Y	UBM	0.41 ± 0.03 mm	N.A.	32.40 ± 3.23
Cao et al. [10]	2013	64	V4	1Y	UBM	0.32 ± 0.15 mm	N.A.	29.1% (>30°) 50.0% (21° to 30°) 11.6% (11° to 20°) 9.3% (<10°)
Lim et al. [11]	2014	18	V4	10 to 51M	UBM	265.27 ± 112.83	N.A.	23.27 ± 8.14
Fernández-Vigo et al. [12]	2016	50	V4c	3M	FD-OCT	380.5 ± 171.8 (nasal) 363.8 ± 167.1 (temporal)	0.13 ± 0.06 (nasal) 0.12 ± 0.06 (temporal)	30.6 6 ± 12.3 (nasal) 30.1 ± 11.9 (temporal)
Fernández-Vigo et al. [13]	2017	54	V4c	2Y	FD-OCT	390.1 ± 166.5 (nasal) 354.1 ± 151.9 (temporal)	N.A.	27.3 ± 8.8 (nasal) 26.8 ± 8.1 (temporal)
Coskunseven et al. [14]	2017	20	V4	1Y	FD-OCT	0.414 ± 0.027 mm (nasal) 0.420 ± 0.041 mm (temporal)	0.168 ± 0.013 (nasal) 0.173 ± 0.014 (temporal)	27.77 ± 1.55 (nasal) 27.85 ± 1.47 (temporal)
Li et al. [15]	2018	201	V4c	6M	UBM	0.21 ± 0.06 mm in Group 1, 0.26 ± 0.09 mm in Group 2 for 3′oclock; 0.25 ± 0.09 mm in Group 1, 0.26 ± 0.06 mm in Group 2 for 9′oclock	N.A.	22.67 ± 6.15 in Group 1, 27.56 ± 8.50 in Group 2 for 3′oclock; 26.30 ± 7.11 in Group 1, 25.54 ± 5.72 in Group 2 for 9′oclock
Current	2020	116	V5	18M	SS-OCT	0.32 ± 0.09 mm	0.12 ± 0.05	29.9 ± 7.3

F/U = follow-up; AOD500 = angle open distance at 500 µm; TISA500 = trabecular-iris space area at 500 µm; TIA500 = trabecular iris angle at 500 µm; Y = year; M = month; UBM = ultrasound biomicroscopy; FD-OCT = Fourier domain optical coherence tomography; SS-OCT = swept source optical coherence tomography; N.A. = not available; Group 1 = 54 eyes with primary iris and/or ciliary body cysts; Group 2 = 147 eyes without primary iris and/or ciliary body cysts.

## Abbreviations

ICL	implantable collamer lens
IOP	intraocular pressure
AS-OCT	anterior segment optical coherence tomography
UBM	ultrasound biomicroscopy
ECD	endothelial cell density
D	diopter
SD	standard deviation
logMAR	logarithm of the minimum angle of resolution
AOD500	angle open distance at 500 µm
TISA500	trabecular-iris space area at 500 µm
TIA500	trabecular iris angle at 500 µm
ANOVA	analysis of variance
